# Prevalence of Trichomoniasis, Vaginal Candidiasis, Genital Herpes, Chlamydiasis, and Actinomycosis among Urban and Rural Women of Haryana, India

**DOI:** 10.1155/2014/963812

**Published:** 2014-10-28

**Authors:** Brij Bala Arora, Megha Maheshwari, Naiya Devgan, D. R. Arora

**Affiliations:** ^1^Department of Pathology, SGT Medical College, Budhera, Gurgaon, Haryana 123505, India; ^2^Department of Microbiology, SGT Medical College, Budhera, Gurgaon, Haryana, India; ^3^Flat No. 218, Sector 23, Pocket 1, Rohini, New Delhi 110085, India; ^4^Department of Gynaecology and Obstetrics, SGT Medical College, Budhera, Gurgaon, Haryana, India

## Abstract

Despite being curable reproductive tract infections (RTIs) including sexually transmitted infections continue to be a major health problem in developing countries. The present study was undertaken to know the prevalence of trichomoniasis, vaginal candidiasis, genital herpes, chlamydiasis, and actinomycosis in rural and urban women of Haryana by using wet mount, PAP smear, and fluorescent microscopic examination. Patients suspected of suffering from bacterial vaginosis were given treatment and were not included in the study. RTIs were seen in 16.6% of urban and 28.7% of rural women. The highest prevalence seen was that of trichomoniasis in both rural (24.2%) and urban (15.7%) women, followed by candidiasis (4.2% in rural and 0.6% in urban women), genital herpes (0.3% in rural and 0.2% in urban women), and chlamydiasis (0.02% in rural and 0.05% in urban women). Pelvic actinomycosis was seen in 1.4% of rural and 0.06% of urban women using intrauterine contraceptive devices. Mixed infection of* Trichomonas vaginalis* with* Candida *spp. was seen in 6.3% of rural women only. It is desirable to have a baseline profile of the prevalence of various agents causing RTIs in a particular geographic area and population which will help in better syndromic management of the patients.

## 1. Introduction

Reproductive tract infections (RTIs) including sexually transmitted infections (STIs) continue to be major health problem in developing countries leading to considerable morbidity. Most of the RTIs are prevalent in India; however, their profile varies with changes in socioeconomic, cultural, geographic, and environmental factors prevalent in different parts of the country. Information regarding the laboratory data on RTIs is lacking due to syndromic diagnosis which is adopted by the clinicians. Lack of adequate laboratory infrastructure, limited resources, associated stigma, and poor attendance of female patients in the RTI/STI clinics are few reasons for lack of RTI data as discussed by Ray et al. [1]. The causes, presenting symptoms, and the perception of symptoms may vary in different populations. The prevalence of different causative agents of RTIs may be different in urban and rural population.

RTIs in many cases are asymptomatic among women, making their detection and diagnosis difficult. Routine Papanicolaou (PAP) smear examination can be very useful in such cases. PAP smear has become a routine procedure for women at their annual gynecologic visit because of its success in the prevention of cervical cancer and precursor lesions as discussed elsewhere [2]. In addition to its primary benefit as a cancer screening test, other benefits of the PAP smear include the detection of cervicovaginal microorganisms. Cytopathologists have observed that alterations in cell morphology that accompany certain viral, trichomonal and monilial infections can be recognized in PAP smear submitted for routine light microscopy. The Papanicolaou smear, which is a cost-effective test, fast and acceptable to most patients, can therefore be used in the diagnosis of genital tract infections. PAP smears are as reliable in the diagnosis of trichomoniasis as either wet film examination or culture. In contrast for detection of candidiasis and genital herpes, PAP smear examination is less sensitive [3]. The present study was undertaken to determine the prevalence of trichomoniasis, vaginal candidiasis, genital herpes, genital chlamydiasis, and actinomycosis infection of the female reproductive tract in urban vis a vis rural population using wet mount, PAP smear, and fluorescent microscopic examination. Patients clinically diagnosed as suffering from bacterial vaginosis were treated on the discretion of gynaecologist and a sample for diagnosis of bacterial vaginosis was not sent to the laboratory. Hence these patients were not included in the study.

## 2. Materials and Methods

A total of 12,622 patients visiting the gynaecology and obstetrics outpatient department, presenting with one or more complaints of RTI, were included in the study, over a time period of six years. Clinical examination of the patients was done for all the patients by the gynaecologist. Patients with normal physiological vaginal discharge were excluded from the study. Patients suspected of bacterial vaginosis were given treatment and were not included in the study. Patients with watery/yellowish/curdy white/purulent discharge were included in the study. Vaginal discharge was collected from all these patients and subjected to wet mount examination for detection of* Trichomonas vaginalis* and* Candida* spp. Cervical specimen was also collected from all the patients using an Ayre's spatula; two smears were prepared from each specimen. One smear was immediately wet fixed in 95% ethanol and stained by Papanicolaou's method and examined microscopically. For detection of* Chlamydia trachomatis* fluorescent antibody staining of the second smear prepared from cervical specimen was also done using fluorescent labeled species specific antibodies and the smears were examined under fluorescent microscope to look for inclusion bodies. The patients were called back for follow-up examination after one month of treatment to look for therapeutic response. All those patients who responded to the specific therapy were included in the study.

**Figure 1:**
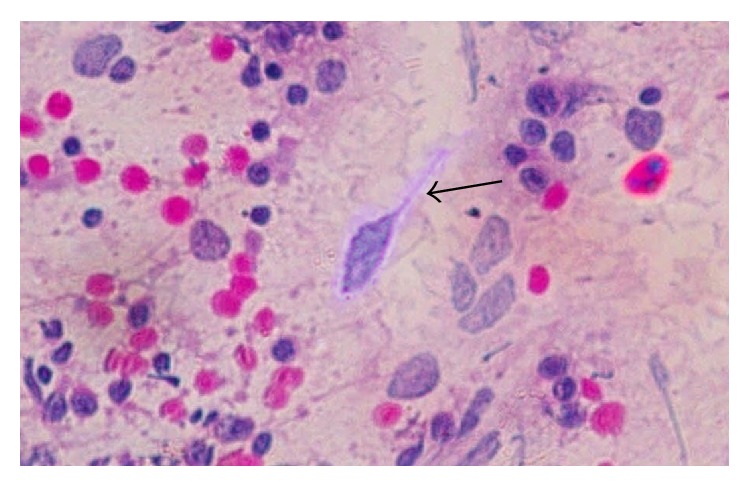
Pear shaped structures with small eccentrically placed nucleus in a background of large number of inflammatory cells along with few squamous cells (Papanicolaou stain ×400).

Trichomoniasis was identified by the presence of motile* Trichomonas vaginalis* in wet smear examination. On PAP smear trichomoniasis was distinguishable by marked nonspecific inflammatory changes in the squamous cells wherein nucleus shows hypertrophy with hyperchromasia and karyorrhexis, varying degrees of cytolysis with cells showing hazy cytoplasmic borders, perinuclear haloes, and vacuolated cytoplasm giving a moth eaten appearance, along with a background of large number of inflammatory cells mainly neutrophils both intact and degenerated.* Trichomonas vaginalis* was identified as green grey pear shaped structure with small eccentrically placed nucleus; remnants of flagella were identified in some cases (Figure 1). A diagnosis of trichomoniasis was made only when trophozoites of* Trichomonas* were seen in the smear irrespective of the degree of inflammatory changes.

**Figure 2:**
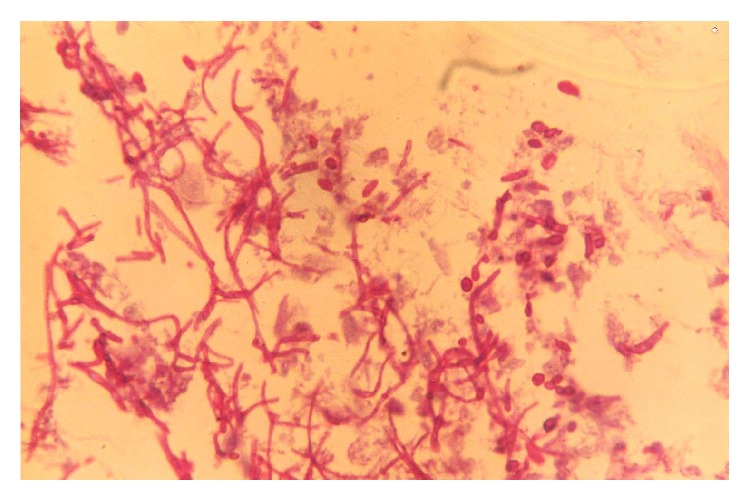
Budding yeast cells with pseudohyphae in a background of some degenerated squamous epithelial cells (Papanicolaou stain ×200).

Candidiasis was identified by the presence of budding yeast cells and pseudohyphae on wet mount. On PAP smear candidiasis was identified by mild degree of inflammatory changes along with presence of budding yeast cells and pseudohyphae (Figure 2). A diagnosis of candidiasis was made only if the presence of fungus was seen with clinical symptoms and vaginal discharge suggestive of vaginal candidiasis. Mere presence of organism in the absence of clinical features was not considered for diagnosis. Culture for further growth and identification of* Candida* spp. was not done.

**Figure 3:**
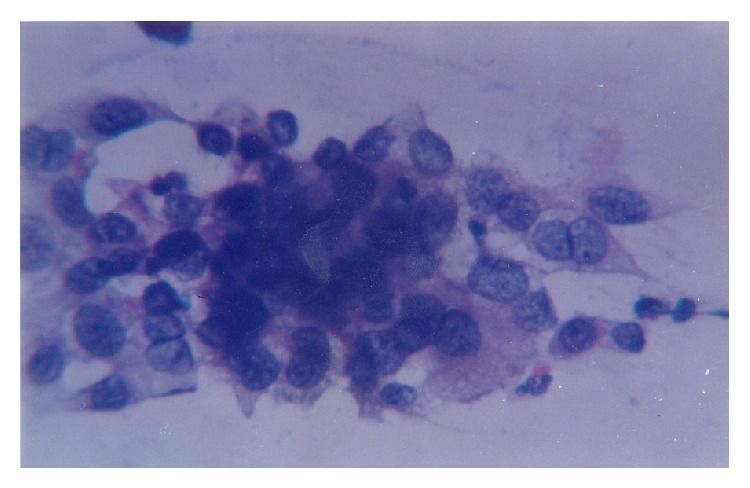
Cells having enlarged nuclei and multinucleated giant cells with nuclear molding and ground glass appearance of some of the cells (Papanicolaou stain ×400).

Herpes simplex virus infection or genital herpes was identified by the presence of cells having enlarged nuclei and multinucleated giant cells with nuclear molding as seen on PAP smear. The nuclear chromatin pattern was lost giving the nucleus a ground glass appearance with condensation of nuclear chromatin at the periphery of the nucleus. Intranuclear inclusion bodies could be seen in some cases (Figure 3).

**Figure 4:**
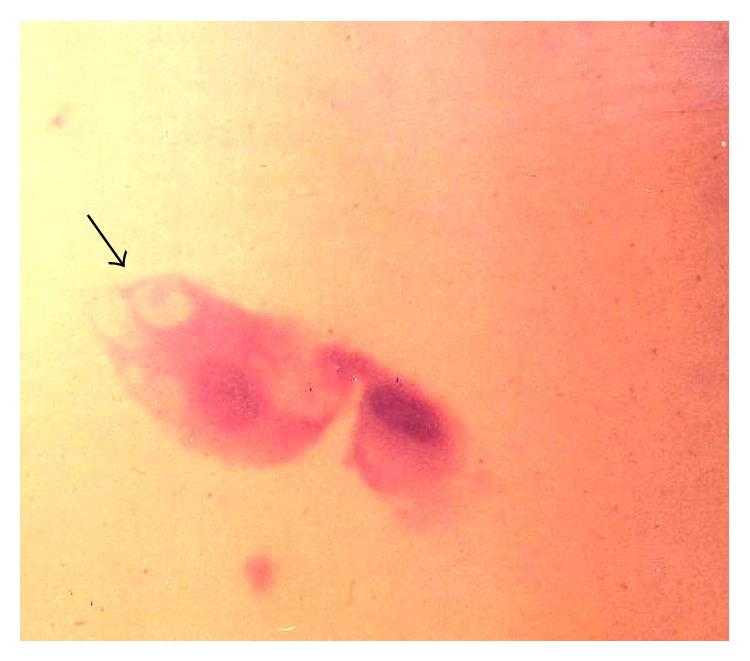
Perinuclear inclusion bodies of* Chlamydia trachomatis* (Papanicolaou stain ×400).

**Figure 5:**
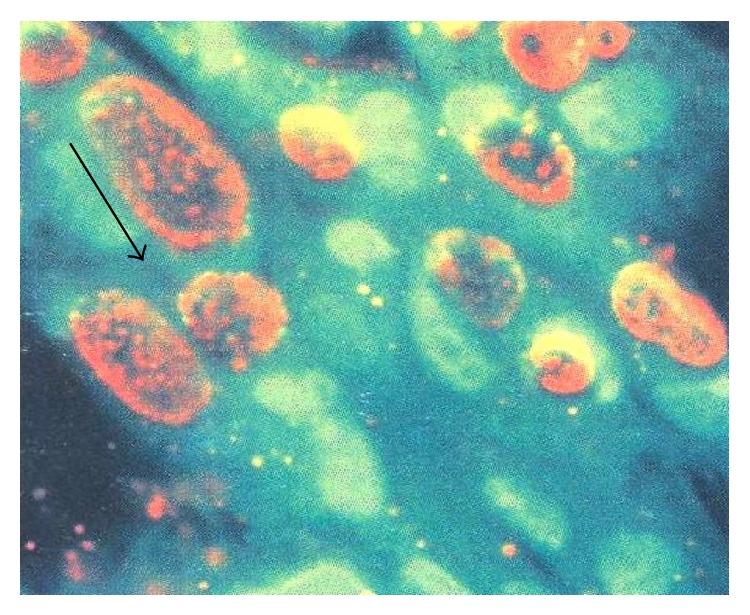
Fluorescent microphotograph of* Chlamydiae trachomatis* showing perinuclear inclusion bodies.

*Chlamydia trachomatis* was identified by the presence of cytoplasmic and perinuclear vacuolisation along with inflammatory cells on PAP smear (Figure 4). Fluorescent microscopy was done using Remel Patho DX* Chlamydia trachomatis* FA Direct Test Kit. On fluorescent microscopy; perinuclear inclusion bodies showing fluorescence were seen in the cells (Figure 5).

**Figure 6:**
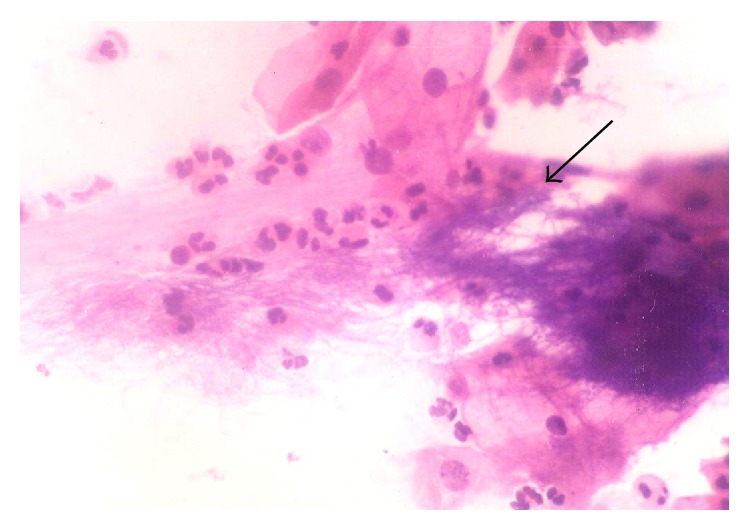
Filamentous organisms resembling* Actinomyces* seen in a background of inflammatory cells along with some intermediate and superficial cells. (Papanicolaou stain ×400.)

Actinomycosis was identified by presence of inflammatory cells and filamentous bacteria resembling* Actinomyces* on PAP smear (Figure 6). Actinomycosis was looked for only in patients using IUCD. Culture confirmation of* Actinomyces* was not done as presence of purulent discharge in IUCD users showing* Actinomyces* like organism was taken as criteria for detection of actinomycosis.

## 3. Results

Of the 12,622 women enrolled for the study, 7950 (63%) were from urban background and 4672 (37%) were from rural background. The age range of urban and rural women was 18−70 years and 15–76 years, respectively. Among the rural women 80% presented with vaginal discharge and the remaining 20% presented with severe backache, though they had vaginal discharge but considered it normal and came to the hospital only when the backache became too severe to tolerate. The urban women were more health conscious and aware of the symptoms and majority (100%) of them presented with complaints of vaginal discharge of short duration. Patients who presented with physiological discharge were excluded from the study on the discretion of gynaecologist. A previous history of RTI was seen in 398/7950 (5%) of urban and 1102/4672 (23.6%) of rural women. The urban women also gave history of coming regularly for yearly routine PAP smear examination after the age of 40, which was not seen in case of rural women. Repeat PAP smear examination was done in patients after one month of treatment and if inflammatory changes were still present they were again given treatment and called back for repeat PAP smear examination after one month. There were 14 such patients who continued to show such changes. Cervical biopsy was done for these patients, which confirmed the presence of neoplastic changes.

The prevalence of RTIs on the basis of prevalence of the organism was 16.6% (1316/7950) in urban and 28.7% (1339/4672) in rural women. Trichomoniasis was the commonest infection followed by vaginal candidiasis, genital herpes, and chlamydiasis in both urban and rural women. While trichomoniasis, candidiasis, and genital herpes were more common in rural women, chlamydiasis was found to be more common in urban women (Table 1). Mixed infection of* Trichomonas vaginalis* was seen with* Candida* spp. in 294 cases (6.3%) among rural women only. Out of the 7950 urban women 1592 (20%) were using intrauterine contraceptive device (IUCD). In contrast only 6% (280/4672) of rural women were using IUCDs as means of contraception. The prevalence of actinomycosis was 0.06% (1/1592) in urban women and 1.4% (4/280) among the rural women using IUCD. Of the 5 patients detected with actinomycosis, sulphur granules were seen in one patient only. The remaining four patients had purulent discharge.

## 4. Discussion

A majority of women continue to suffer from RTIs leading to complications like pelvic inflammatory disease (PID), infertility, postabortal, puerperal sepsis, chronic pelvic pain, ectopic pregnancy, and cervical cancer. RTIs in many cases are asymptomatic among women, making their detection and diagnosis difficult. The various RTIs may be ulcerative or nonulcerative. The most common presenting complaint of RTI in women is vaginal discharge as reported by various studies as discussed elsewhere [4, 5]. In the present study also vaginal discharge was the most common presenting complaint in both urban and rural populations. However, the complaint of backache was found in the rural women. In a study done by Nandan et al. backache was more commonly seen with rural women as compared to the urban women [6]. One explanation for this, as was seen in our study also, could be that a mild vaginal discharge was considered as normal by our rural population and they did not visit the hospital. When the discharge became severe and was accompanied by severe backache they came to visit the OPD for the backache problem.

The prevalence of RTI was higher in rural population (28.7%) than urban population (16.6%). A study done by Nandan et al. in Agra also found a high prevalence of STI in rural population (49%) than urban population (27%) [6]. The reasons for this high prevalence in rural women could be lack of awareness, poor literacy rates, low socioeconomic status, poor personal hygiene and most importantly poor treatment seeking behavior as also described by Ray et al. [1].

Very high prevalence of RTI in the study population was seen by Kosambiya et al. [5]. In the study they also found that the prevalence was higher in urban population (69%) as against (53%) the rural population and the prevalence of trichomoniasis was highest at 18% in urban population and 22% in rural population on the basis of wet mount. Various studies have found trichomoniasis as the commonest infection among the various reproductive tract infections. The prevalence of trichomoniasis in other studies was 23.1%, 32.8%, and 41%, respectively, as discussed elsewhere [3, 7, 8]. However, there are studies in which a very low prevalence of trichomoniasis was seen and the prevalence ranges from 2.1 to 6% as discussed elsewhere [1, 9].

The prevalence of candidiasis in the present study was 0.6% in urban and 4.2% in rural population. Similar results were seen in a study by García et al. who found 4.5% prevalence of candidiasis among women suffering from RTIs [10]. In a study from Surat the prevalence of candidiasis was found to be 14% among urban women and 12% among rural women suffering from RTIs including STIs [4]. Few other studies have shown a high prevalence of candidiasis at 16% and 40%, respectively, as discussed elsewhere [11, 12].

Mixed infection of trichomoniasis with candidiasis in the present study was seen only in the rural population and the prevalence was 6.3%. According to a study done in Delhi by Ray et al., the prevalence of mixed infection of trichomoniasis and candidiasis was 3% among rural and 5.3% among urban women [1]. Very low coinfection rates (0.23%) were reported by Angelique, whereas several studies done on RTIs have not witnessed mixed infection as discussed elsewhere [2, 5, 6]. A study done in Iran has shown very high rates of mixed infection (64.8%) of trichomoniasis and candidiasis [13].

The prevalence of genital herpes (1.6 and 2.5 per thousand in urban and rural women, resp.) in our study was very low as compared to another study from Delhi which reported a prevalence of 28.6% [4]. A study done in Dhaka reported a prevalence of 12% [14].

The prevalence of chlamydiasis was very low in the present study, both among rural and urban population. In a study done in Lebanon no case of chlamydiasis was seen [11]. However, a prevalence of genital chlamydiasis ranging from 3% to 17% has been reported as discussed elsewhere [12, 15].

Female genital* Actinomyces *infection is relatively rare, although strongly related to long-lasting intrauterine contraceptive device (IUCD) application. Actinomycosis is not routinely considered a causative agent of RTI as it is seen with prolonged usage of plastic IUCDs. Studies indicate that* Actinomyces israelii* may infect 1.6%–11% of IUCD users worldwide and are mostly related to plastic IUCDs without hormonal or metal load as discussed elsewhere [16]. Low prevalence rate of actinomycosis among urban women in the present study was due to frequent change of IUCDs as compared to rural women.

There are wide variations in the reported prevalence of various RTIs, depending on the population studied and geographic areas and the diagnostic methods used. The study highlights the need for laboratory investigations so that baseline data is available on the exact prevalence of the disease in the concerned populations. The PAP smear has been widely accepted as a model screening test for cervical cytology. It is cost effective, fast, and convenient test and is acceptable to most patients. This test can also be used for detection of genital tract infections. An experienced cytologist can make a reliable diagnosis of trichomoniasis; however, for other RTIs like candidiasis and genital herpes it is less sensitive. The utility of PAP smear in detecting RTIs has been recognized by the United Nations Population Fund (UNFPA) which has recommended that PAP smear programmes should integrate with the RTI detection clinics [17]. Such integration will be useful especially in resource poor settings where one visit and one test will solve two purposes. In addition wet smear examination and Gram stain of vaginal discharge can be done to support the diagnosis. It is recommended that both partners should be treated simultaneously for RTI to prevent retransmission of infection.

Knowledge of etiologies common to a particular population or area will help in the syndromic management of cases where laboratory investigations are not feasible. The World Health Organization (WHO) has placed emphasis on syndromic approach for case measurement and management, particularly in high-prevalence areas having inadequate laboratory facilities, trained staff, and transport facilities.

## Figures and Tables

**Table 1:** Prevalence of various causative agents of genital infection in urban and rural women.

Population	Urban (*n* = 7950)		Rural (*n* = 4672)	
	Number of positive	Percentage	Number of positive	Percentage
*Trichomonas *	1249	15.71	1130	24.18
*Candida *	50	0.62	196	4.19
HSV	13	0.16	12	0.25
*Chlamydia *	04	0.05	01	0.02
Mixed infection of *Trichomonas *and *Candida *	Nil	Nil	294	6.3
*Actinomyces *in IUCD users	01/1592	0.06	04/283	1.41
